# Comparative Transcriptome Analysis of Raccoon Dog Skin to Determine Melanin Content in Hair and Melanin Distribution in Skin

**DOI:** 10.1038/srep40903

**Published:** 2017-01-18

**Authors:** Zhanyu Du, Kai Huang, Jiaping Zhao, Xingchao Song, Xiumei Xing, Qiong Wu, Linbo Zhang, Chao Xu

**Affiliations:** 1 Key Laboratory of Special Economic Animal Genetic Breeding and Reproduction, Ministry of Agriculture, State Key Laboratory of Special Economic Animal Molecular Biology, Institute of Special Animal and Plant Sciences of Chinese Academy of Agricultural Sciences, Juye Street NO. 4899 130112, Changchun, China; 2 College of Life Science, Jilin Agricultural University, Xincheng Street NO. 2888 130118, Changchun, China; 3 Beijing Gene-Health Huachuang Biotech Co., Ltd, Xueqing Rode NO. 9 100089, Beijing, China; 4 Key Laboratory of Farm Animal Genetic Resources and Germplasm Innovation, Ministry of Agriculture (nzdsys2016-3), Yuangmingyuan West-Rode NO. 2 100193, Beijing, China.

## Abstract

The raccoon dog (*Nyctereutes procyonoides*) is an important canid fur-bearing animal species worldwide. Chinese raccoon dogs that present a white mutation, especially those with a white coat. Exploring melanin biosynthesis in the hair and skin of raccoon dogs is important for understanding the survival and evolutionary mechanisms of them. In this study, we measured the content of melanin in the hair of two types of raccoon dog and generated stained slices of skin tissue. The results indicated that melanin biosynthesis occurs in the wild-type (W) and white-type (B) raccoon dog skin, although less melanin is produced in B skin. We then sequenced the skin transcriptomes of W and B, compared the similarities and differences in expressed genes. A comparison of the gene expression showed 60 up-regulated genes and 127 down-regulated genes in B skin. We analyzed the unigenes and pathways related to the melanogenesis pathway and found that *TYR, TYRP1, MC1R, SLC24a5, SLC45a2* and *OCA2* were significantly down-regulated in B skin and these results were verified via qRT-PCR. We surmised that the phenotypic characteristics of the white mutation might be caused by the reduced expression of these genes and this finding provides new insights for future experiments in raccoon dogs.

The raccoon dog (*Nyctereutes procyonoides*) is a canid with a high reproductive rate, short generation time, high population turnover and generalized diet^1^,^2^,^3^. The native distribution area of this mammal includes southeastern Russia, the eastern provinces of China, northern Vietnam, and Japan^4^,^5^. The Chinese wild-type raccoon dog (Nyctereutes procyonoides procyonoides), which has a mixed coat color of black-to-brown with grey hairs^6^, is an important fur-bearing animal species worldwide^7^,^8^. The first Chinese white mutation raccoon dog was identified in 1970, and other than its white coat, its physical characteristics are essentially the same as those of the wild-type raccoon dog. The Institute of Special Animal and Plant Sciences of the Chinese Academy of Agricultural Sciences (CAAS) introduced the white raccoon dog variety in 1982 and renamed it the “Jilin white raccoon” in 1990.

Mammalian coat color is decided by two types of melanin, eumelanin and pheomelanin, which present differences in their ratios and transformations. The absence and synthesis of melanin is the basis for the formation of the mammalian coat color^9^. Melanosomes are intracellular organelles that are uniquely generated by pigment cells in the skin and eye, where they function to synthesize and store melanin pigments^10^. Eumelanosomes are large (~0.9 × 0.3 μm) and ellipsoidal and have a highly ordered glycoprotein matrix that is integral to the production of the black or brown-colored eumelanin pigments, whereas red or yellow pheomelanins are produced within smaller and spherical (~0.7 μm diameter) pheomelanosomes that are composed of a loosely aggregated and disordered glycoprotein matrix^9^. Based on these characteristics, Ito and Wakamatsu^11^ studied melanin in humans, mice and other species to investigate the relationship between hair and skin melanin changes and skin color or hair color. Aliev *et al*.^12^ studied the melanin content of different sheep strains and different sheep coat colors. Sponenberg *et al*.^13^ studied the melanin content of different horse coat colors. Cecchi *et al*.^14^,^15^ investigated melanosomes and differences in the melanin contents of different llama hair fibers.

Hair is a derivative of skin and exits the surface of mammalian skin via hair follicle growth and development. Mammalian skin is covered with colorful hair because of hair follicle morphogenesis. According to the period of hair follicle morphogenesis, hair is divided into primary hair follicles (PHFs) and secondary hair follicles (SHFs). In raccoon dogs and other furry mammals, the hair growing out of the PHFs is called guard hair and the hair growing out of the SHFs is called fluff. Although different mammals have various types of guard hair and fluff, the basic structures of their follicles are equivalent.

Follicular pigmentation is a result of structural and functional interactions between follicular melanocytes, matrix keratinocytes and dermal papilla fibroblasts^16^. This tripartite system is described as the hair melanin unit or follicular melanin unit. The process of hair pigmentation includes the melanogenic activity of follicular melanocytes, the transfer of melanin granules into keratinocytes and the formation of pigmented hair shafts^17^,^18^. In the pigmentation that determines hair color, the following elements are involved: melanocortin receptor 1 (MCR1) and its α-MSH, adrenocorticotropic hormone (ACTH), receptor c-Kit and its ligand SCF, endothelins, different neurotransmitters, cytokines, growth factors and other similar regulators for epidermal melanogenesis control^17^,^19^.

Melanogenesis is a biochemical pathway responsible for melanin synthesis^20^. The availability of substrates and the function of melanogenesis enzymes determine the types of melanins produced. Tyrosine changes into a polymer of melanin, which is a mixture of the pigments eumelanin (black-brown) and pheomelanin (yellow-red), under the influence of its enzymes, such as tyrosinase (TYR) and tyrosine-related protein 1 (TYRP1) and 2 (TYRP2)^16^. Melanosome development requires tyrosinase and the two tyrosinase-related proteins TYRP1 and TYRP2. Of these three enzymes, tyrosinase is crucial to melanogenesis, and it is synthesized on the ribosomes of the Rough Endoplasmic Reticulum and transported to the Golgi complex, where it undergoes glycosylation, which is an essential process for its normal structure and functions^21^.

Transcriptional profiling is a powerful approach for the global identification of genes and their functional expression in various tissues, including skin^22^. Limited information is currently available on the differences in the transcriptome profiles of skin associated with coat color in fiber-producing species. To investigate genes that play important roles in coat color regulation in raccoon dog skin and gain insights into the molecular mechanisms responsible for the biochemistry of the skin and fibers (including the pigmentation) of the raccoon dog, we investigated the transcriptome profiles of the skin of raccoon dog presenting wild-type versus white coat colors using high-throughput deep RNA sequencing. The results provided novel insights into the differences in gene expression associated with coat color and identified the key genes implicated in the melanogenesis pathway.

## Results

### Determination of melanin content

According to the method of determining melanin content, we produced a standard curve line of the melanin content and obtained the regression equation of the melanin concentration related to absorbance at 500 nm: C (μg/mL) = 234.82 A500–22.198 (r = 0.9995). The measured absorbance of the melanin content at 500 nm and 650 nm in the wild-type (W) and white-type (B) raccoon dogs is shown in Table 1.

### Melanin observations in histological skin sections

**Figure 1:**
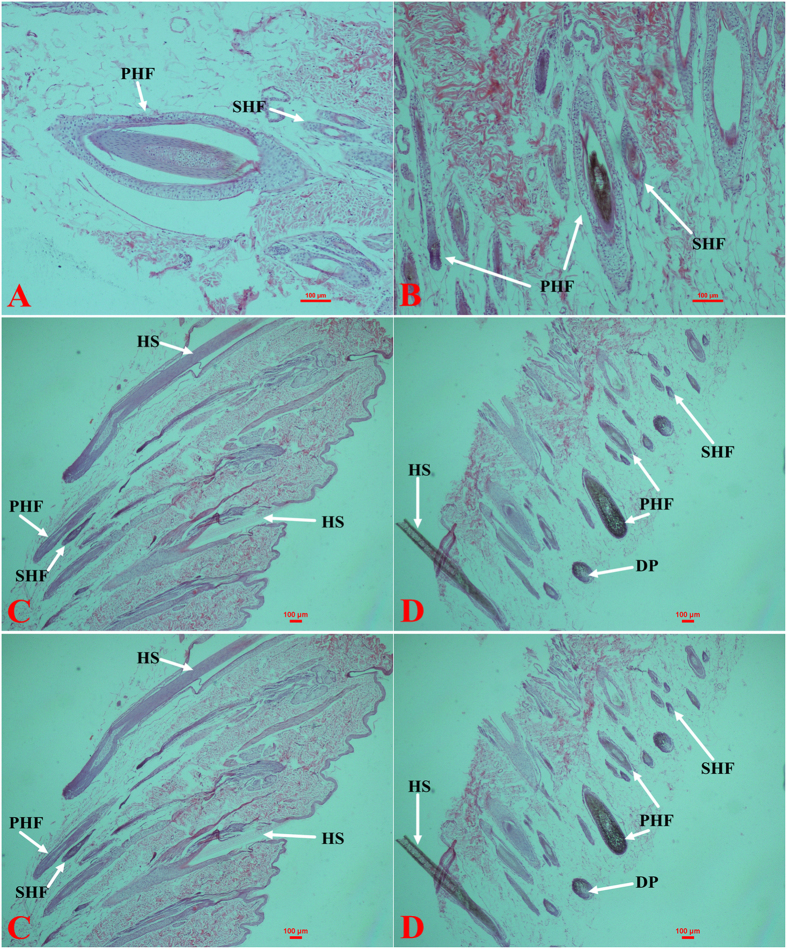
H&E staining of raccoon dog skin tissue. Longitudinal sections of HSs in W (B × 100, D × 40) and B (A × 100, C × 40) skin and transverse sections of HSs in W (F × 40) and B (E × 40) skin. PHF: primary hair follicle; SHF: secondary hair follicle; DP: dermal papilla; and HS: hair shaft.

**Figure 2:**
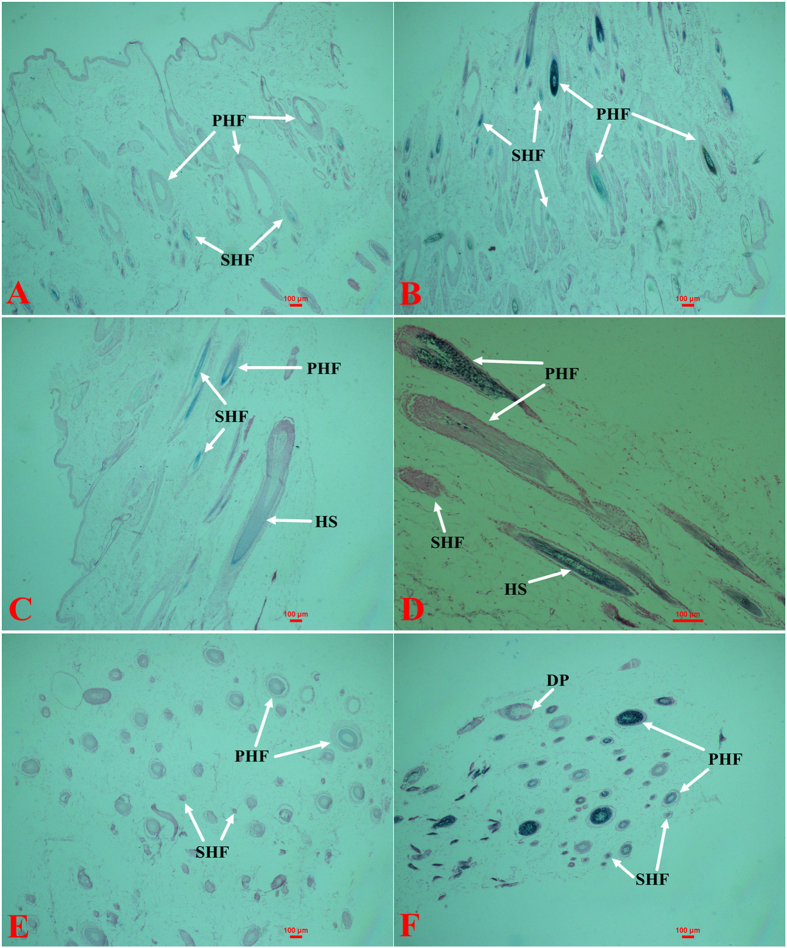
Ferrous sulfate staining of raccoon dog skin tissue. Longitudinal sections of HSs in W (B × 40, D × 40) and B (A × 40, C × 40) skin and transverse sections of HSs in W (F × 100) and B (E × 40) skin. PHF: primary hair follicle; SHF: secondary hair follicle; DP: dermal papilla; and HS: hair shaft.

The W skin follicular morphology indicates that 4 to 5 SHFs occur around PHFs, which are 4 or 5 times larger than SHFs (Fig. 1b,d and f, Fig. 2b and f), and the density of the PHFs and SHFs is higher in the W skin than in the B skin (Figs 1 and 2). Melanin mainly occurs in the hair shaft (HS) (Fig. 1c and d, Fig. 2c and d). The PHFs were filled with melanin granules, whereas the SHFs were not (Figs 1 and 2). An average of 2 or 3 SHFs were observed around a PHF (Fig. 1a,c and e) and fewer melanin granules were observed in the PHFs and SHFs of B skin (Fig. 2a,c and e).

### Assembly of unigenes

After the raw reads were filtered, 50,464,640 clean reads with 51.10% GC and 49,709,974 clean reads with 50.67% GC were obtained from the W and B skin, respectively. These clean reads were assembled into unigenes, with 98,505 and 102,897 unigenes obtained from the W and B skin, respectively. The unigenes were annotated using the following databases: NR, NT, Swiss-Prot, KEGG, COG and GO. Table 2 shows the number of unigenes annotated with each database.

### Differentially expressed genes in raccoon dog skin

**Figure 3:**
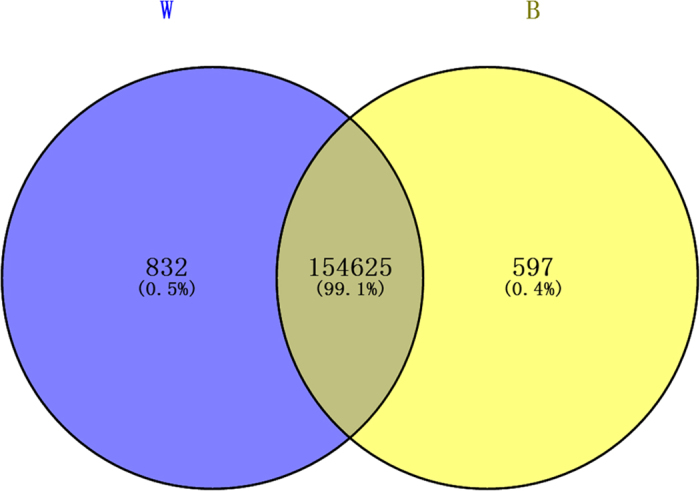
Differentially expressed genes that are unique or shared between the W and B skin. W refers to the wild-type skin raccoon dog group. B refers to the white-type skin raccoon dog group. The numbers in each section of the figure indicate the number of differentially expressed genes in the indicated comparison.

A comparison of the gene expression in the W and B skin showed that a total of 187 unigenes were significantly differentially expressed (|log2 (fold change)| > 1, P value ≤ 0.05), with 60 up-regulated genes and 127 down-regulated genes in the B skin. Moreover, 154,625 unigenes were common expressed in both the W and B skin. In addition, 832 and 597 unigenes were uniquely expressed in the W and B skin, respectively. All of the differentially expressed genes are illustrated in Fig. 3.

### KEGG pathway analysis

**Figure 4:**
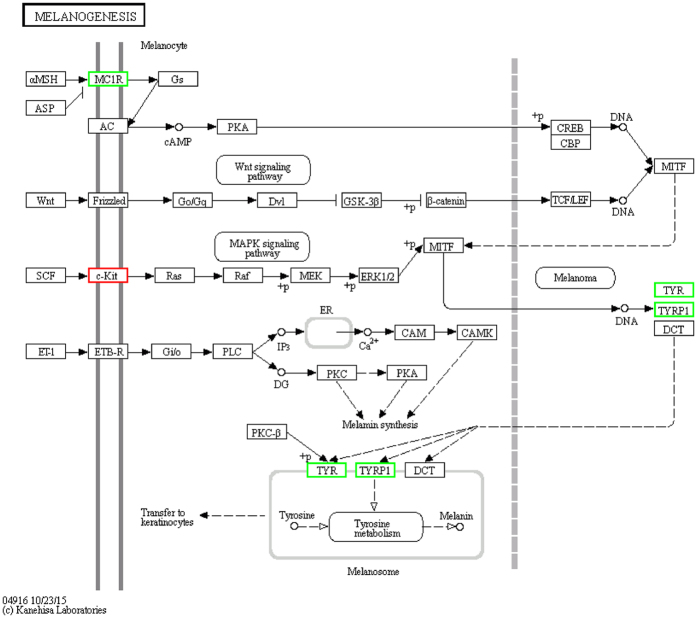
Differentially expressed skin color genes identified in the analyzed raccoon skin and their involvement in the melanogenesis pathway. The genes with a red frame are up-regulated in the white-type skin compared with the wild-type skin. The genes with a green frame are down-regulated in the white-type skin compared with the wild-type skin.

**Figure 5:**
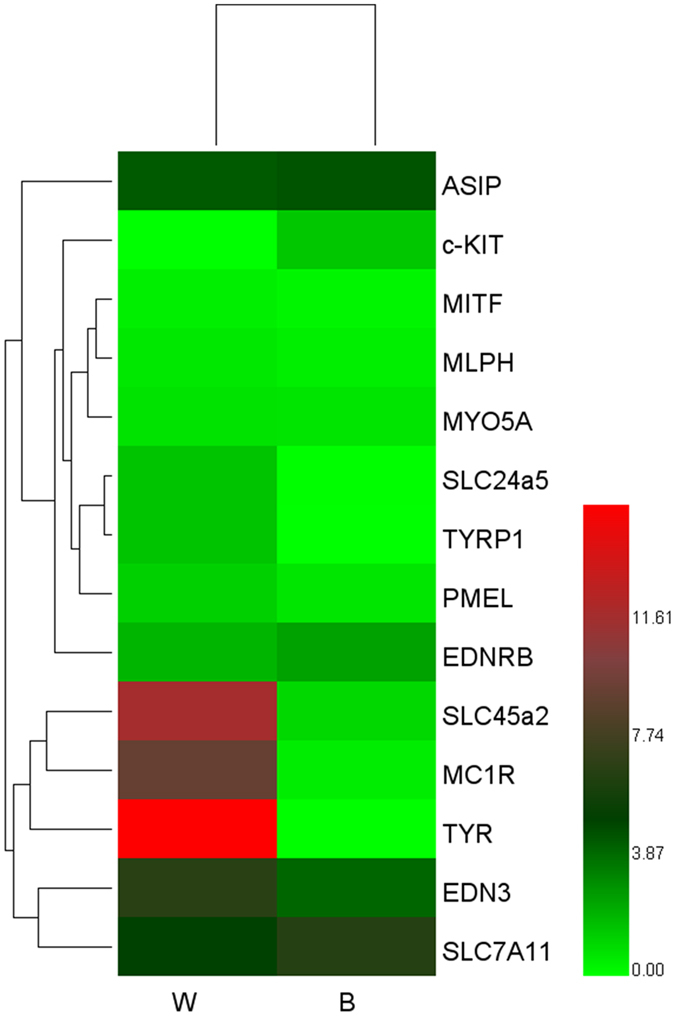
A heat-map exploring the differences in gene expression between the W and B skin. Different colors represent different expression levels, and a darker color represents higher expression and a greater log2 (FPKM) value.

Of the 50,831 known differentially expressed genes in the W and B skin, 112 had a specific KEGG pathway annotation. Of these KEGG pathway annotated genes, 14 were down-regulated in the W skin. These down-regulated genes are mainly involved in Organismal Systems and Metabolism. Two and 6 differentially expressed genes were involved in the tyrosine metabolism and melanogenesis pathways, respectively. The enriched GO terms for the genes identified in the raccoon dog skin transcriptome are related to pigmentation and melanogenesis, and their relative expression in the W versus B skin is shown in Fig. 4. All of the unigenes were mapped to 259 KEGG pathways, including the following signaling pathways: MAPK, calcium, Wnt and VEGF. Other pathways associated with skin pigmentation include the melanogenesis, melanoma and tyrosine metabolism pathways, although the current study focused on the melanogenesis pathway. We created a heat map of the 14 differentially expressed genes that play an important role in animal pigmentation and are involved in the melanogenesis pathway (Fig. 5).

### Validation of the differentially expressed mRNA in the raccoon dog skin

**Figure 6:**
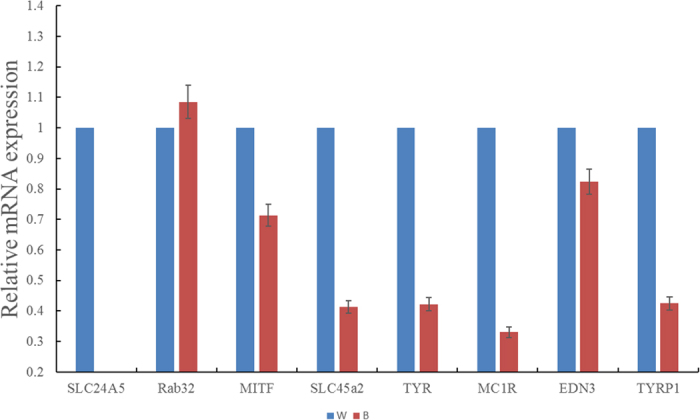
Quantification of the mRNA levels of the RNA which extracted from the skin of W (Wild-type raccoon dogs) or B (White-type raccoon dogs). The bars represent the adjusted ct values; therefore, higher values represent higher mRNA levels.

Among these 187 significantly differentially expressed unigenes, we selected *ASIP, KIT, MITF, SLC24a5, SLC45a2, TYR, MC1R, TYRP1 EDN3* and *Rab32* which main related to melanin synthesis to validated the expression levels though the method of real-time qPCR. The results indicated that significant differences did not occur in the expression of ASIP, KIT, MITF, SLC24a5, SLC45a2, EDN3, and Rab32 between the W and B type (Fig. 6), although the expression of MC1R, TYR and TYRP1 was significantly down-regulated in the B skin (P value ≤ 0.05). The results of the qPCR were consistent with the RNA sequencing.

## Discussion

Mammalian hair color is directly determined by the melanin content. In the normal trunk skin of adult mice, mature melanocytes are only present in the hair follicles (HFs)^23^,^24^ during anagen, which is the stage of the hair cycle when the active growth and pigmentation of hair occurs^25^,^26^. Therefore, the best stage of the hair cycle for determining the melanin content is the telogen stage^27^ because melanin is not produced in the HF^28^. We measured the melanin content of hair in the W and B types and found that the hair of B appears white and has less melanin. We then used ferrous sulfate and hematoxylin-eosin (H&E) to stain the tissue sections of the W and B skin to observe the distribution of melanin, and the results indicated that most melanin is concentrated in the shaft of the hair follicle. Most hair follicles of B skin have a small accumulated amount of melanin and less SHFs, which is also stained by ferrous sulfate. The results indicated that melanin biosynthesis also occurred in the B skin, which produced less melanin.

After analyzing the transcriptome results, we observed gene expression differences between the W and B types in the melanogenesis pathway, such as in the *MC1R, TYR, TYRP1* and *KIT* genes. Both melanin biosynthesis and accumulation occur on the melanosome, and melanocortins (MSH) bind to the G-protein coupled receptor MC1R, thereby resulting in up-regulated cAMP levels, which in turn trigger the eumelanin biosynthesis process. The synthesis of eumelanin is then catalyzed successively by *Tyr, DCT, TYRP1* and *PMEL* (also known as *gp100* or *SILV*)^29^. All four of the enzymes involved in melanogenesis are transcriptional targets of MITF and present an identical TCATGTG sequence in their proximal promoters that is close to the start of transcription^30^. The rate-limiting initial steps for melanin synthesis (e.g., hydroxylation of tyrosine to L-DOPA and oxidation of L-DOPA to DOPAquinone) are catalyzed by the pigment cell-specific enzyme tyrosinase (*TYR*). Subsequent spontaneous oxidation and isomerization steps yield a mixture of eumelanin intermediates. Two other enzymes, DOPAchrome tautomerase (*DCT*) and tyrosinase-related protein 1 (*TYRP1*), influence the nature of the intermediates and properties of the eumelanins^20^. Research on *KIT*^31^ in raccoon dog reveals that the B Chinese raccoon dog version of *KIT* lacks transcriptional activity. We surmised that the expression of *KIT* is not an influencing factor in the B mutation from the W. Moreover, our transcriptome results showed that the expression of the *TYR, TYRP1* and *MC1R* genes was significantly down-regulated in B, which was verified via qRT-PCR, although *MITF* was not down-regulated.

Tyrosinase processing and trafficking is regulated by the organellar pH, and tyrosinase is inactive at pH < 6.0^32^. The membrane-associated transporter protein *SLC45A2* is responsible for the maintenance of melanosome pH by removing H^+^ in exchange for Na^+^. The Na^+^ ions are then cycled back out of the melanosome by the cation exchanger *SLC24A5*^33^. Early stage melanosomes are highly acidic^34^; therefore, OCA2-mediated Cl^-^ transport shifts the endolysosomal pH toward the neutral values required for optimum tyrosinase activity and melanin synthesis^35^. Our results showed that the expression of *SLC24A5, SLC45A2* and *OCA2* was down-regulated in the B skin. We presumed that the internal melanosome environment may not be in balance with the pH because of the variations in gene expression, particularly because TYR activity was not the highest. Future studies should investigate the internal environment of melanosomes and the activity of TYR in the W skin compared with the B skin.

Rab32 regulates a critical step in the trafficking of melanogenic enzymes, particularly TYR, from the trans-Golgi network (TGN) to melanosomes. Wasmeier *et al*.^36^ analyzed the effect of Rab32-specific small interfering RNA in cht cells and observed a dramatic loss of pigmentation. In Rab32-deficient cells, tyrosinase appears to be mistargeted and degraded after it exits the TGN. However, both the transcriptome and qRT-PCR results showed that the expression of Rab32 was not significantly different between the W and B skin. A recent study on feline coat patterns proposed that the patterned coat is sustained by the comparatively high expression of EDN3 in dark marking regions, stimulating region-specific eumelanogenesis via the EDNRB pathway^37^. However, in our study, the expression of EDN3 was not significantly different between the W and B skin.

## Methods

### Ethics statement

This study was approved by the Institutional Animal Care and Use Committee (IACUS) of the Chinese Academy of Agricultural Sciences (CAAS). The collection of raccoon dog samples was permitted by the Institute of Special Economic Animal and Plant Sciences of the CAAS in Jilin Province, China, and all of the sampling procedures complied with the guidelines of the IACUS on the care and use of animals for scientific purposes.

### Determination of melanin content

Three wild-type (W) and three white-type (B) male raccoon dogs were selected from the experimental base of the Chinese Academy of Agricultural Sciences in Jilin Province. After administering anesthesia, 2 cm × 2 cm of hair coat was cut from the right-hand side of the animals’ backs. The samples were washed, air dried, and sorted, and the guard hair and fluff were placed in valve bags. We improved upon a previous method by transforming the guard and fluff hair into powder, weighing 5 mg of the powder into EP tubes and adding 0.5 mL Soluene^®^-350 (PerkinElmer) which mix with water at 9:1. We then vortexed and centrifuged the samples for 10 seconds and maintained the temperature at 100 °C for 30 minutes to 1 hour using a thermostat. An appropriate shock was performed to promote hair dissolution. Subsequently, we assessed 200 μL of this solution to determine the absorbance at 500 nm and 650 nm. The solution was weighed according to melanin standards using a precision balance, and Soluene^®^-350 liquid was added to a stock solution at a concentration of 2 mg/mL. The solution was then diluted to various concentrations, and we assessed 200 μL to determinate the absorbance at 500 nm. A standard curve was then generated to obtain the equation.

### Skin staining

The W or B skin samples were fixed with 4% paraformaldehyde for 3 days and then dehydrated through a graded series of ethanol and embedded in paraffin. Six-millimeter sections were cut using a microtome (Leica, Germany) and then stained with ferrous sulfate and hematoxylin-eosin (H&E).

### Total RNA isolation, library construction and sequencing

Total RNA was isolated using TRIzol Reagent (Invitrogen) according to the manufacturer’s instructions from each skin sample after pulverizing the sample in liquid nitrogen. The quality and concentration of total RNA were determined using an Agilent 2100 Bioanalyzer (Agilent). The RNA samples were stored at −80 °C for subsequent library construction and sequencing. Three RNA libraries were constructed for each skin sample. The mRNA was fragmented and reverse transcribed using random primers. Second-strand cDNAs were synthesized using RNase H and DNA polymerase I. The double-stranded cDNAs were then purified using the QiaQuick PCR extraction kit. The required fragments were purified via agarose gel electrophoresis and enriched via PCR amplification. Finally, the amplified fragments were sequenced using an Illumina HiSeq™ 2000 system (Beijing Gene-Health Huachuang Biotech Co., Ltd, China) according to the manufacturer’s specifications.

### Mapping reads to the reference genome

The original sequencing image data were transformed into sequence data via base calling, which is defined as raw data or raw reads stored in the fastq format. The raw reads of all six samples were pre-processed by removing the adaptor reads with more than 5% unknown nucleotides. Low-quality reads (percentage of low-quality bases with quality values ≤5 in more than 50% of a read) were also removed. Mismatches of no more than two bases were allowed in the alignment, and uniquely mapped reads were obtained.

### Expression annotation

For the gene expression analysis, the number of uniquely matched reads was calculated and normalized to the FPKM (fragments per kilobase of exon model per million mapped reads). The expression levels of each gene between the two groups were compared, and the expression difference was determined using DESeq as described by Abders and Huber^38^. The P values corresponded to differential gene expression at statistically significant levels^39^. The FDR (false discovery rate) was used to determine the P value threshold. DEGs were defined at FDR ≤0.05 and an absolute log2Ratio value ≤1.

The union set of all DEGs was used for further expression pattern analyses. STEM (Short Time-series Expression Miner, v1.3.8) was used to profile the gene set into eight expression patterns. Cluster profiles with a q value ≤ 0.05 were considered significantly expressed. The GO (gene ontology) annotations were analyzed using Blast2GO software (version 2.3.5) (https://www.blast2go.com/). Functional classifications of the DEGs was performed using WEGO software. The KEGG (Kyoto Encyclopedia of Genes and Genomes) pathway annotations were conducted using Blastall software against the KEGG database (http://www.kegg.jp/).

### Confirmation of RNA-seq results with qPCR

First-strand cDNA was generated from 1 μg total RNA isolated from the skin samples using the Superscript™ First-strand Synthesis System (Invitrogen). To confirm the transcriptomic analysis results, eleven genes that were consistent with those derived from sequencing were selected and subjected to qPCR (quantitative reverse transcription PCR); these genes included Rab32, MITF, SLC24a5, SLC45a2, TYR, MC1R, EDN3, and TYRP1. ACTB (beta-actin) was chosen as an internal reference gene because it presented equivalent FPKM values among the three stages. The qPCR was conducted on a CFX Connect Real-time PCR Detection System (Bio-Rad) using SYBR Premix Ex Taq (TaKaRa). The qPCR was run under the following conditions: 50 °C for 2 min and 95 °C for 10 min; and then 45 cycles of 95 °C for 15 s, 60 °C for 1 min, and 72 °C for 45 s. Each qPCR analysis was performed in triplicate. The relative gene expression levels were calculated using the 2^−ΔΔCt^ method^40^. The primers used for the qPCR are listed in Supplemental Table 1.

## Additional Information

**How to cite this article**: Du, Z. *et al*. Comparative Transcriptome Analysis of Raccoon Dog Skin to Determine Melanin Content in Hair and Melanin Distribution in Skin. *Sci. Rep.* **7**, 40903; doi: 10.1038/srep40903 (2017).

**Publisher's note:** Springer Nature remains neutral with regard to jurisdictional claims in published maps and institutional affiliations.

## Supplementary Material

Supplementary Table 1

## Figures and Tables

**Table 1:** Melanin content data.

Melanin content (μg/mg)	W		B	
	Guard hair	Fluff	Guard hair	Fluff
All	26.922	24.704	4.228^*^	3.822^*^
Eumelanin	12.001	9.255	1.323^*^	1.248^*^
Pheomelanin	21.173	15.449	2.905^*^	2.574^*^

^*^P value < 0.005.

**Table 2:** Databases for the annotation of unigenes.

Sample	NR	NT	Swiss-Prot	KEGG	COG	GO	ALL	
W	57845	97224	54758	47715	27966	44279	98505	
B	58960	101582	55726	48389	28225	44908	102897	
