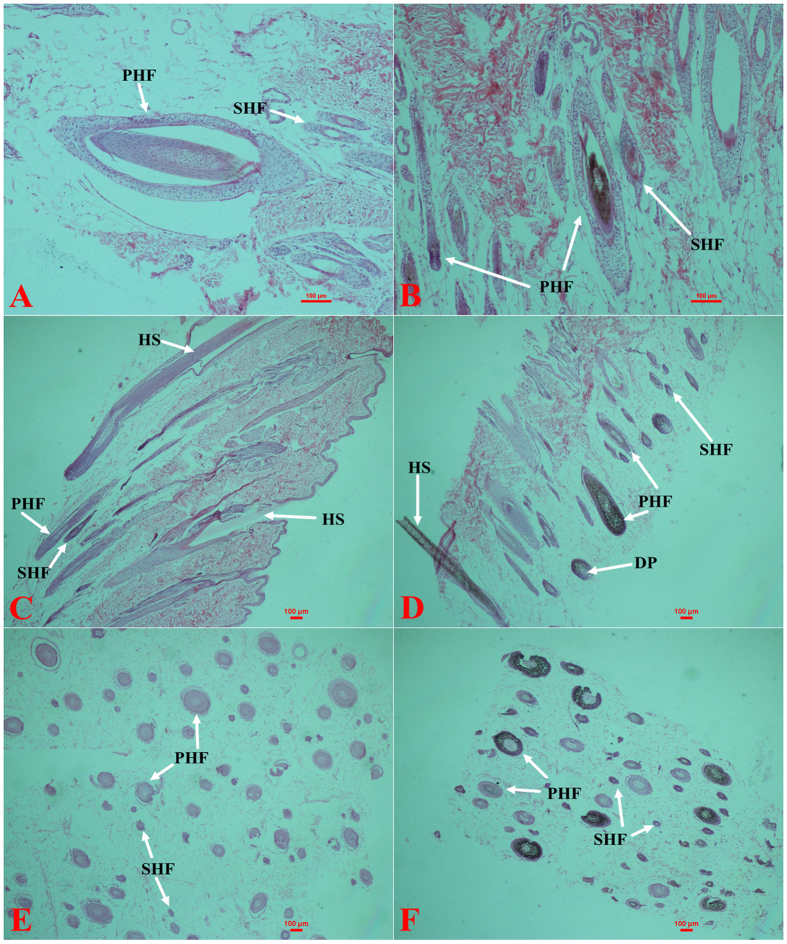# Corrigendum: Comparative Transcriptome Analysis of Raccoon Dog Skin to Determine Melanin Content in Hair and Melanin Distribution in Skin

**DOI:** 10.1038/srep44344

**Published:** 2017-03-16

**Authors:** Zhanyu Du, Kai Huang, Jiaping Zhao, Xingchao Song, Xiumei Xing, Qiong Wu, Linbo Zhang, Chao Xu

Scientific Reports 7: Article number: 4090310.1038/srep40903; published online: 01 18 2017; updated: 03 16 2017

This Article contains errors in Figure 1 where Figure 1C and Figure 1D were inadvertently duplicated, and Figure 1E and Figure 1F were omitted. The correct Figure 1 appears below as Figure 1.

## Figures and Tables

**Figure 1:**