# Supplementary material for: Comparative Transcriptome Analysis of Raccoon Dog Skin to Determine Melanin Content in Hair and Melanin Distribution in Skin
Source: Sci Rep. 2017 Jan 18;7:40903. doi: 10.1038/srep40903 (PMC5241637; doi:10.1038/srep40903)
Supplement: Supplementary Table 1 [file srep40903-s1.doc]

**Comparative Transcriptome Analysis of Raccoon Dog Skin to Determine Melanin Content in Hair and Melanin Distribution in Skin**

Zhanyu Du^1,2^, Kai Huang^3^, Jiaping Zhao^1^, Xingchao Song^1^, Xiumei Xing^1^, Qiong Wu^1^, Linbo Zhang^2^, Chao Xu^1,4*^

^1^Key Laboratory of Special Economic Animal Genetic Breeding and Reproduction, Ministry of Agriculture, State Key Laboratory of Special Economic Animal Molecular Biology, Institute of Special Animal and Plant Sciences of Chinese Academy of Agricultural Sciences.

^2^College of Life Science, Jilin Agricultural University.

^3^Beijing Gene-Health Huachuang Biotech Co., Ltd

^4^Key Laboratory of Farm Animal Genetic Resources and Germplasm Innovation, Ministry of Agriculture (nzdsys2016-3)

**^*^****Corresponding author**

**PhD** Chao Xu

Institute of Special Animal and Plant Sciences of Chinese Academy of Agricultural Sciences.

Juye street of No. 4899 in Changchun, Jilin Province, People’s Republic of China.

[xuchao@caas.cn](mailto:xuchao@caas.cn)

Supplemental Table 1. List of primers used in qRT-PCR validation of DEs identified by RNA sequencing

| Gene | Primers | Sequences (5' to 3') | Product Size (bps) | Contig name |
| --- | --- | --- | --- | --- |
| *Rab32* | Forward | CCGATTCCTGCTGTCCTCTTA | 80 | Unigene74941_All |
|  | Reverse | AACTGGTCCATCTGAGGAGGATT |  |  |
| *MITF* | Forward | CTTGTACCACTGTCCTGACTTTCG | 140 | CL6924.Contig3_All |
|  | Reverse | ATAAAACCACCCCCGAGGAT |  |  |
| *SLC24a5* | Forward | AACAGGAGCGCACAGATGGAT | 150 | CL24.Contig2_All |
|  | Reverse | CACCTGCAACATCCTGAGACAT |  |  |
| *SLC45a2* | Forward | TGGCATTGCAGGGAGAAATGC | 150 | Unigene47730_All |
|  | Reverse | ACAGGAAAGCAGTCCATCCAG |  |  |
| *TYR* | Forward | ATTGGCAGCTTTATCCATGGA | 80 | Unigene67052_All |
|  | Reverse | GACTCCCCTCCTCTGCTGATG |  |  |
| *MC1R* | Forward | TGGACCGCTACCTCTCCATCTT | 142 | Unigene18194_All |
|  | Reverse | GCCGTGTGACTGTAGTAGGCAAT |  |  |
| *END3* | Forward | ATCACATCCCGCCCTCACT | 80 | Unigene46244_All |
|  | Reverse | GCCTTGCCTTTTTGGTCTTTC |  |  |
| *TYRP1* | Forward | GGCCAGGACAGGAAAGCTTT | 80 | Unigene18357_All |
|  | Reverse | TAGGTGGTATCTGTGCCATGTGA |  |  |
| *β-actin* | Forward | CATGGGCCAGAAGGACTCCTA | 148 | Unigene14320_All |
|  | Reverse | GCGCAGCTCGTTGTAGAAGGT |  |  |
